# An Analysis of the Readability of Online Sarcoidosis Resources

**DOI:** 10.7759/cureus.58559

**Published:** 2024-04-18

**Authors:** Shahmeen Irshad, Nasir Asif, Usman Ashraf, Hamzah Ashraf

**Affiliations:** 1 Internal Medicine, Richmond University Medical Center, New York, USA; 2 Medicine, Rutgers University, Newark, USA; 3 Medicine, Rutgers University, New Brunswick, USA

**Keywords:** internet, medical education, health literacy, readability, sarcoidosis

## Abstract

Introduction

Sarcoidosis is an inflammatory disease characterized by the formation of noncaseating granulomas in multiple organ systems. The presentation can vary widely; although some patients with sarcoidosis can be asymptomatic, sarcoidosis can also present in others with symptomatic multiorgan system involvement. Considering the potential severity of the disease, patients need to be well-informed about sarcoidosis to better manage their health. This study aims to assess the readability levels of online resources about sarcoidosis.

Methods

We conducted a retrospective cross-sectional study. The term "sarcoidosis" was searched online using both Google and Bing to find websites written in English. Each website was categorized by type: academic, commercial, government, nonprofit, and physician. The readability scores for each website were calculated using six different readability tests: the Flesch-Kincaid reading ease (FKRE), Flesch-Kincaid grade level (FKGL), Gunning fog score (GFS), Simple Measure of Gobbledygook (SMOG), automated readability index (ARI), and Coleman-Liau index (CLI). FKRE gives a score that corresponds to the difficulty of the text, while the remaining tests give a score that corresponds to a grade level in terms of reading ability. A one-sample t-test was used to compare all test scores with the national recommended standard of a sixth-grade reading level. Our null hypothesis was that the readability scores of the websites searched would not differ statistically significantly from the sixth-grade reading level and that there would be no significant differences across website categories. To evaluate the difference between the categories of websites, ANOVA testing was used.

Results

Thirty-four websites were analyzed. Each of the six readability tests for the websites had an average score, which corresponded to being significantly harder to read than the nationally recommended sixth-grade reading level (p<0.001). None of the mean readability scores showed a statistically significant difference across the five different website categories.

Conclusions

This is the first study, to our knowledge, to examine the readability of online English resources on sarcoidosis and calculate standardized readability scores for them. It implies that the online English material for sarcoidosis is above the health literacy recommended reading levels for patients. There is a need to simplify the material to be easier to read for patients.

## Introduction

Sarcoidosis is an inflammatory disease characterized by the formation of noncaseating granulomas in multiple organ systems [1]. The prevalence of this malady is approximately 10-20 per 100,000 individuals [1]. Sarcoidosis affects Black Americans more frequently than White Americans and typically occurs in younger patients [2]. It can range from being asymptomatic in some patients to symptomatic multiorgan involvement in other patients [3]. It is theorized that a combination of genetic and environmental factors are responsible, with possible infectious and autoimmune involvement, but the exact cause remains undefined [4]. Pulmonary fibrosis is the most common cause of death from sarcoidosis in Western countries [5]. Sarcoidosis is typically treated with corticosteroids and immunosuppressive agents, such as methotrexate, azathioprine, or an anti-tumor necrosis factor medication [6]. The mortality rate from this disorder is 1%-8% [7].

Given the potential severity of the disease, it is important for patients to be compliant with medication. Research has shown that patients who are more educated about their chronic disease tend to be more compliant with medication than patients who are not [8]. This makes sense, as they have a better understanding of what the medication does to combat the disease and the risks of not taking the medication [8]. Patients with sarcoidosis who are more adherent to their medication have also been found to have a higher quality of life compared to those who are not [9]. Patients who are more "health-literate" have been reported to have higher rates of medication adherence compared to those who are less "health-literate" [10]. Health literacy is defined as "the personal, cognitive, and social skills that determine the ability of individuals to gain access to, understand, and use information to promote and maintain good health" [11].

Internet healthcare resources play a critical role in patient information and decision-making [12]. These can include websites that give patients more information about their diseases regarding identification and management. Sarcoidosis is no exception, as there are plenty of articles online discussing its symptoms when patients should suspect it and talk to a physician, certain treatments, and more. One study found that more than half of people looking for health information had their health behavior influenced by online resources [13]. Because of the great influence the Internet can have over prospective patients, it is important to ensure that these sources are easily accessible. However, many online health sources can be difficult to understand because of the way they are written with complex terminology and sentence structure [14].

The average adult reads at about an eighth-grade level [15]. Meanwhile, the average Medicare beneficiary is only capable of a fifth-grade reading level [16]. Given the low literacy rates in America, the National Institutes of Health (NIH), the American Medical Association (AMA), and the United States Department of Health and Human Services (USDHHS) have all recommended that health education materials for patients be written at a sixth-grade reading level or below [17,18].

Given this fact, we wished to discover the readability level of articles on sarcoidosis commonly found on the Internet and whether they matched the official recommendations.

## Materials and methods

This study did not require Institutional Review Board approval, and there was no patient involvement. Based on the methodology by Mc Carthy et al., we performed a retrospective cross-sectional study [19]. Their methodology consisted of searching for "slipped upper femoral epiphysis" on different search engines, collecting the websites from the first two pages of search results, eliminating websites that met their exclusion criteria, and then analyzing the readability scores of the websites left using WebFx.com [19].

In March 2023, "sarcoidosis" was inputted into Google and Bing. Table 1 shows the number of results for each Internet search engine. The inclusion criteria included the first 25 websites in English from each search engine; prior research has shown that people are unlikely to look at search results beyond the first 25 [20]. This corresponds to roughly the first two pages of search results on an Internet search engine. Prior readability studies have also used this cutoff [21,22].

**Table 1:** Results by search engine

Search engine	Hits returned
Google	24,500,000
Bing	359,000

Exclusion criteria were applied that prohibited duplicate websites, medical journals, pages requiring login information, and websites that were unable to be analyzed for readability. This is seen in Table 2. Medical journals were excluded as they were considered too complex for a normal person to understand, following the reasoning in prior readability studies [19].

**Table 2:** Summary of websites excluded

Criteria	Website (n)
Websites found	50
Duplicates	8
Medical journal	2
Login required	3
Unable to be analyzed	3
Websites included	34

Afterwards, the websites to be used in the analysis were classified according by type, drawing on the methodology used by a previous readability study [19]. The five categories included academic, commercial, government, nonprofit, and physician. “Academic” included websites that were owned or associated with a university. “Commercial” included websites that had advertisements. “Government” were websites associated with governments of countries or government agencies. “Nonprofit” involves websites operated by nonprofit groups or NGOs (nongovernmental organizations). “Physician” refers to websites owned by individual physicians or physician groups (e.g., American Academy of Dermatology). These categories are shown in Table 3.

**Table 3:** Websites by category

Category	Websites (n)
Academic	8
Commercial	8
Government	8
Nonprofit	7
Physician	3

WebFx.com is a free website that can calculate how readable, or how easy to read, other websites are. This tool was used to analyze and collect data on the websites we chose. Notably, as indicated in Table 2, three websites were excluded from the final analysis because they could not be interpreted by WebFx.com to provide us with data for them. WebFx.com works by providing scores for six readability tests: Flesch-Kincaid reading ease (FKRE), Flesch-Kincaid grade level (FKGL), Gunning fog score (GFS), Simple Measure of Gobbledygook Index (SMOG), Coleman-Liau index (CLI), and automated readability index (ARI). These tests are elaborated on in Table 4, adapted from Zhou et al., which offers a brief description of each test and the formula used to calculate the readability score [23].

**Table 4:** Information on readability tests Adapted from Zhou et al. [23]

Test name	Description	Formula
Flesch-Kincaid reading ease (FKRE)	Created by Rudolf Flesch. Used to predict the readability of a text. Uses the number of syllables, words, and sentences in its equation.	206.835 - 1.015 (words/sentences) - 84.6(syllables/words)
Flesch-Kincaid grade level (FKGL)	Predicts the reading grade level of written material. Developed by J Peter Kincaid for the US Navy. Uses the number of syllables, words, and sentences in its equation.	0.39 (words/sentences) + 11.8 (syllables/words) - 15.59
Gunning fog score (GFS)	Predicts the reading grade level of written material. Invented by Robert Gunning. Uses the number of sentences, words, and complex words (defined as words with three or more syllables) in its equation.	0.4 [(words/sentences) + 100(complex words/words)]
Simple Measure of Gobbledygook (SMOG)	Predicts the reading grade level of written material. Designed by G Harry McLaughlin. Uses the number of sentences and complex words (defined as words with three or more syllables) in its equation.	1.043√(complex words x 30 / number of sentences) +3.1291
Automated readability index (ARI)	Predicts the reading grade level of written material. Developed by RJ Senter and EA Smith for the US Air Force. Uses the number of words, sentences, and characters in its equation.	4.71 (characters/words) + 0.5(words/sentences) -21.43
Coleman–Liau index (CLI)	Predicts reading grade level of written material. Created by Meri Coleman and TL Liau. Uses the average number of letters per 100 words and the average number of sentences in its equation.	0.0588 (average number of letters per 100 words) - 0.296(average number of sentences per 100 words) - 15.8

The FKRE is one of the most used measures of readability; a higher score corresponds to higher readability [24]. Table 5, adapted from Spadaro et al., shows FKRE values and their corresponding readability levels, with higher scores corresponding to easier readability [24]. For example, a score of 60 would be appropriate for a 9th or 10th-grade reading level. For the remaining five tests, their scores are supposed to correlate to grade-level indicators. Thus, a score around seven would be suitable for a seventh-grade reading level, while a score of nine would be appropriate for a ninth-grade reading level [23,25]. As such, for these five tests, in contrast to FKRE, a lower score corresponds to higher readability [25].

**Table 5:** Flesch-Kincaid reading ease (FKRE) scale interpretation Adapted from Spadaro et al. [24]

FKRE score	Grade level
90-100	5
80-90	6
70-80	7
60-70	8-9
50-60	10-12
30-50	College
0-30	College graduate

Microsoft Excel version 16.36 was used to collect the data, which was then analyzed using Statistical Product and Service Solutions (SPSS, version 25; IBM SPSS Statistics for Windows, Armonk, NY) and RStudio (version 1.2.5042; RStudio Team, Boston, MA). RStudio was used for calculations of one-sided, one-sample t-tests; all other remaining statistical tests were performed on SPSS, which is unable to calculate one-sided, one-sample t-tests. Significance was set for P values less than 0.05. ANOVA testing was used to compare the means and analyze differences among the five types of websites. 

One-sample t-tests were performed using a sixth-grade reading level as the standard, which corresponds to a score of 80 for FKRE and 6 for non-FKRE tests (FKGL, GFS, SMOG, ARI, CLI). This is because the AMA and NIH have recommended that education materials for patients should not be written with a readability score higher than sixth-grade level [17,18].

Our null hypothesis was that the mean FKRE scores of the websites would be greater than or equal to 80 (so at sixth-grade reading level or easier), while the mean scores for non-FKRE tests (i.e., FKGL, GFS, SMOG, ARI, CLI) would be less than or equal to 6 (also at sixth-grade reading level or easier).

Our alternative hypothesis was that the mean FKRE score of the websites would be less than 80 (indicating a reading level harder than sixth grade), while the mean non-FKRE test scores for the websites would be greater than six (also indicating a reading level harder than sixth grade).

## Results

Twenty-five websites were found on both the first two pages of Bing and Google, for a total of 50 websites. These websites are listed in Table 10 as supplementary material. Eight websites were duplicates between the two search engines, meaning 42 unique websites. Eight websites were excluded as they were either medical journals, required logins, or could not be analyzed. Table 2 summarizes the websites excluded. A total of 34 websites were used in the analysis, with 11 unique for Google, 15 unique for Bing, and 8 for both search engines. Table 3 demonstrates the included websites separated into five categories. The most common categories were academic (n=8; ≈ 24%), commercial (n=8; ≈ 24%), and government (n=8; ≈ 24%), followed by nonprofit (n=7; ≈ 21%), and lastly physician (n=3; ≈ 9%). The mean values for each readability test are presented in Table 6. All readability test scores were above the sixth-grade reading level.

**Table 6:** Readability test mean values FKRE: Flesch-Kincaid reading ease; FKGL: Flesch-Kincaid grade level; GFS: Gunning fog score; SMOG: Simple Measure of Gobbledygook; ARI: automated readability index; CLI: Coleman-Liau index

	FKRE	FKGL	GFS	SMOG	ARI	CLI
Mean +/- SD	46.9 +/- 13.7	8.7 +/- 2.2	9.1 +/- 2.7	7.0 +/- 1.1	7.2 +/- 1.7	15.1 +/- 4.5

Table 7 shows the results of the t-test for FKRE. It was a one-sample one-sided t-test and was conducted against a value of 80 with a significance level of 0.05. The P value was <0.05, indicating that the mean FKRE value of the websites analyzed on sarcoidosis is statistically significantly less than the recommended FKRE value of 80. Since it was a one-sided t-test looking to see if the mean value of FKRE would be less than 80, we only get the upper bound of the 95% confidence interval. The upper bound of the 95% confidence interval is 50.88. This means that we are 95% confident that the mean value of the FKRE of the websites is lower than 50.88. 

**Table 7:** One-sample t-test comparing mean readability score with recommended standards for FKRE

Type of test	Mean value	Significance (one-tailed P value)	95% confidence interval for upper
Flesch-Kincaid reading ease	46.9	<0.001	50.8834

Table 8 shows the results of the t-tests for the non-FKRE reading tests. All five of these t-tests were one sample, one-sided t-tests, conducted against a value of 6 with a significance level of 0.05. Since our alternative hypothesis was that mean non-FKRE test scores for the websites would be greater than 6, we only obtained the lower bound of the 95% confidence interval. For all five t-tests, the P value is <0.05, indicating that each reading test value analyzed for the websites on sarcoidosis is statistically significantly greater than the recommended value of 6. The upper bound of the 95% confidence interval is also listed for each test in Table 8, indicating that we are 95% confident that the mean value for each of those tests for the websites is greater than the number listed.

**Table 8:** One-sample t-test comparing readability score with the recommended standard for non-FKRE tests

Type of test	Mean value	Significance (one-tailed P value)	95% confidence interval for lower
Flesch-Kincaid grade level	8.7	<0.001	8.024691
Gunning fog score	9.1	<0.001	8.308503
SMOG index	7.0	<0.001	6.734341
Automated readability index	7.2	<0.001	6.742142
Coleman-Liau Index	15.1	<0.001	13.74603

Table 9 presents the results of the one-way ANOVA, which compares each readability test’s mean value across the six different website categories. None of the readability tests showed a statistically significant difference across the website categories, as the P value was >0.05 for each. Therefore, the different website categories were not statistically significantly different concerning their readability for any of the readability tests.

**Table 9:** One-way ANOVA comparison of each readability test across categories

Type of test	Category	(Mean value)				P value
	Academic	Commercial	Government	Nonprofit	Physician	
FKRE	51.7	49.3	47.1	41.1	40.8	0.57
FKGL	7.8	7.9	8.4	10.5	9.6	0.10
GFS	8.5	8.2	9.1	10.1	11.0	0.48
SMOG	6.9	6.8	7.3	6.8	8.1	0.35
ARI	6.9	6.7	7.3	7.7	8.1	0.69
CLI	15.2	15.6	15.8	13.1	15.9	0.81

**Figure 1:**
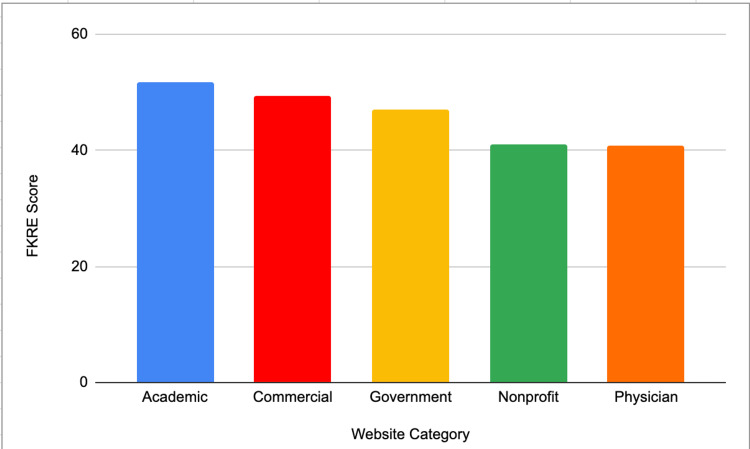
FKRE scores across website categories

**Figure 2:**
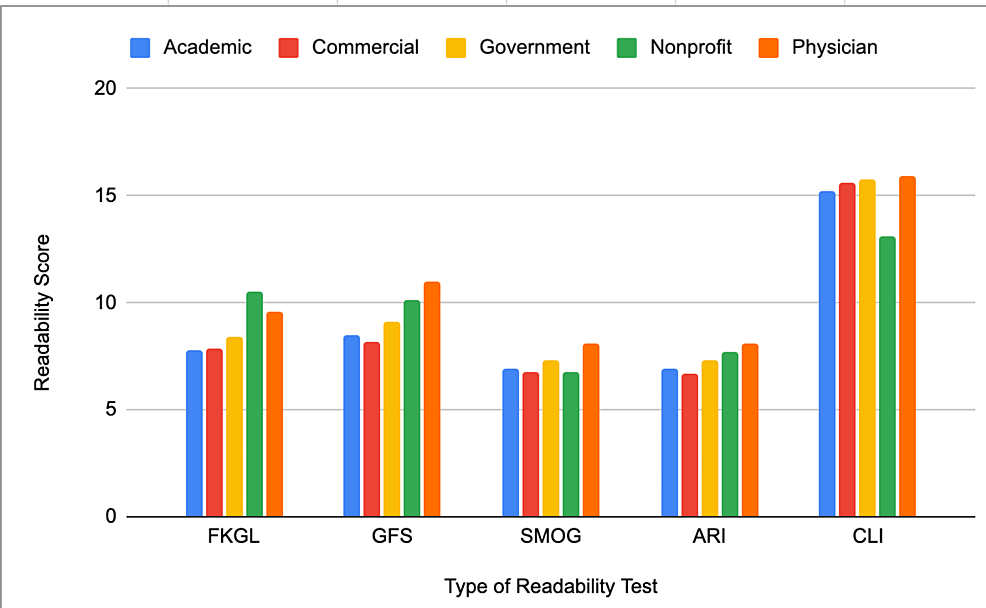
Non-FKRE scores across website categories

The mean readability values for FKRE are presented in Figure 1. The mean readability values for non-FKRE tests are shown in Figure 2. All categories had average readability scores above the recommended sixth-grade reading level.

## Discussion

This study shows that current online resources for sarcoidosis are inadequate for patient education. As was mentioned earlier, an FKRE value of 80 corresponds to the government-recommended sixth-grade reading level [17,18]. The mean FKRE value of the websites in our analysis was 46.9. Using a one-sample, one-tailed t-test, this mean value was statistically significantly lower than a score of 80 (p<0.001). This indicates that these websites are more difficult to read than a sixth-grade reading level. We found similar results for the other reading tests. As mentioned, the non-FKRE tests are grade-level indicators, meaning that their scores correspond exactly to the reading level of the text. For the non-FKRE tests (FKGL, GFS, SMOG, ARI, CLI), when comparing their respective mean values (8.7, 9.1, 7.0, 7.2, 15.1) using a one-sample, one-tailed t-test, they were all statistically significantly greater than the government-recommended sixth-grade reading level (p<0.001). This demonstrates that websites about sarcoidosis found on Google and Bing are not written at the optimal level for patient education and health literacy regarding this disease.

The disappointing results for readability in online sarcoidosis material appear to match similar results for other immune-mediated diseases. Studies on the readability of online resources on lupus, systemic sclerosis, and Sjogren’s syndrome reveal readability numbers that are well beyond the recommended standards [26-28]. In fact, the FKGL scores for systemic sclerosis (11.5) and Sjogren’s Syndrome (12.21) are even higher than the FKGL scores we found for sarcoidosis (8.7) in our analysis [26,28]. This readability issue is not isolated to this field of medicine. Various other disciplines across the medical field also struggle with online resources being above the recommended patient reading levels, including urology, neurosurgery, pediatrics, and more [19, 29-32].

The ANOVA revealed that there were no statistically significant differences with respect to readability among the different website types. This shows that there is no particular resource type that patients can rely on for education on sarcoidosis. Unfortunately, ll of the website categories are above the recommended reading levels set by expert organizations. While one could potentially excuse academic and physician websites for having high readability scores, as their target audience instead of patients likely includes other clinicians or scientists trying to refresh their knowledge about a disease, this reasoning does not apply to commercial, government, and nonprofit websites. The creators of these websites should try to make their websites easier to read for patients, requiring a less complex lexicon. Moreover, even the academic and physician websites could also try their best to simplify language and avoid jargon so that if a patient were to use their websites, those patients could be more likely to understand more.

There are very real and crucial consequences from these findings. The end result is a missed opportunity to tackle health literacy across these fields and regarding these diseases. Low health literacy has led to a cost of anywhere between $50 billion and $73 billion per year [33]. Health literacy is the single best predictor of health status, so patients need to have a good understanding to have the best medical outcome [33]. Patients with limited health literacy have considerable gaps in their knowledge; therefore, they struggle to follow self-care and medical advice [34]. Health literacy also enhances patient-physician communication, which can substantially improve compliance and clinical outcomes [35]. A lack of comprehensible resources can cause issues with the patient-physician relationship and the patient’s understanding of their ailment. Thus, boosting health literacy by having more easy-to-read material on the internet is imperative to help lead to more favorable outcomes for patients, including for patients managing sarcoidosis. This could potentially be achieved by using simpler language, avoiding jargon, and other means.

Limitations of this study include the fact that only English-language websites were analyzed. Census data indicate that approximately 22% of nearly 42 million Spanish-speaking Americans either did not speak English well or at all [36]. This means that there are 9-10 million Americans who cannot get health information from English websites; rather, they use websites written in Spanish for their health information needs. This is worth investigating in a future research project. Additionally, the use of readability formulas, while convenient to use, are not definitive tools for measuring readability. These tools may have shortcomings, as some of the formulas used the number of syllables to gauge the difficulty of reading. For example, text containing words such as “dermal” and “pleural” would be considered easier to understand than text with words such as “steroids” and “disease.” The former, while shorter, are part of the medical lexicon that the average individual is less likely to know, while the latter are more mainstream and likely to be known.

## Conclusions

This is the first study to evaluate the readability of content on sarcoidosis on websites written in English. It shows significant evidence that the material available on the internet is beyond the recommended literacy level for patients. Interventions such as intentionally simplifying the language and avoiding jargon should be undertaken to increase readability and simplify information for patient education. This could lead to improved disease outcomes, as patients would be better equipped to make appropriate healthcare decisions. Further studies should be conducted to examine the readability of online resources written in Spanish, given the prevalence of Spanish-speaking populations.

## Appendices

**Table 10:** Supplementary material: list of websites found on Google and Bing

Google Links	Bing Links
https://www.lung.org/lung-health-diseases/lung-disease-lookup/sarcoidosis/learn-about-sarcoidosis#:~:text=Sarcoidosis%20is%20an%20inflammatory%20disease,skin%2C%20heart%20and%20nervous%20system	https://www.mayoclinic.org/diseases-conditions/sarcoidosis/symptoms-causes/syc-20350358
https://www.mayoclinic.org/diseases-conditions/sarcoidosis/symptoms-causes/syc-20350358	https://my.clevelandclinic.org/health/diseases/11863-sarcoidosis
https://my.clevelandclinic.org/health/diseases/11863-sarcoidosis	https://www.nhlbi.nih.gov/health/sarcoidosis
https://www.nhs.uk/conditions/sarcoidosis/	https://www.healthline.com/health/sarcoidosis
https://www.nhlbi.nih.gov/health/sarcoidosis	https://entirelyhealth.com/conditions/sarcoidosis/symptoms-and-diagnosis-of-sarcoidosis/?utm_source=bing&utm_medium=cpc&utm_campaign=Sarcoidosis%20Disease%20Sy-D&utm_content=1362295852648493&utm_term=sarcoidosis%20disease&msclkid=9d2dfbad46321b8f19749fbb7dd2ac32
https://en.wikipedia.org/wiki/Sarcoidosis	https://healthprep.com/conditions/symptoms-of-sarcoidosis/?utm_source=bing&utm_medium=search&utm_campaign=566520064&utm_content=1177578007558373&utm_term=sarcoidosis&msclkid=222a5718a44b18fdb76c330e9fabb327
https://medlineplus.gov/sarcoidosis.html	https://www.mayoclinic.org/diseases-conditions/sarcoidosis/diagnosis-treatment/drc-20350363
https://www.stopsarcoidosis.org/what-is-sarcoidosis/	https://www.webmd.com/lung/arthritis-sarcoidosis
https://www.hopkinsmedicine.org/health/conditions-and-diseases/pulmonary-sarcoidosis	https://www.hopkinsmedicine.org/health/conditions-and-diseases/pulmonary-sarcoidosis
https://emedicine.medscape.com/article/301914-overview	https://www.yalemedicine.org/conditions/sarcoidosis
https://www.webmd.com/lung/arthritis-sarcoidosis	https://www.nhlbi.nih.gov/health/sarcoidosis/symptoms
https://www.mountsinai.org/health-library/diseases-conditions/sarcoidosis	https://www.verywellhealth.com/sarcoidosis-overview-5097521
https://www.healthline.com/health/sarcoidosis	https://www.stopsarcoidosis.org/what-is-sarcoidosis/
https://www.thoracic.org/patients/patient-resources/resources/sarcoidosis-pt-1.pdf	https://www.aad.org/public/diseases/a-z/sarcoidosis-symptoms
https://www.thoracic.org/patients/patient-resources/resources/what-is-sarcoidosis.pdf	https://my.clevelandclinic.org/health/diseases/23485-cardiac-sarcoidosis
https://nyulangone.org/conditions/sarcoidosis/types	https://www.aafp.org/pubs/afp/issues/2016/0515/p840.html
https://www.betterhealth.vic.gov.au/health/conditionsandtreatments/sarcoidosis	https://www.mayoclinic.org/diseases-conditions/sarcoidosis/doctors-departments/ddc-20350365
https://www.merckmanuals.com/professional/pulmonary-disorders/sarcoidosis/sarcoidosis	https://dermnetnz.org/topics/sarcoidosis
https://facty.com/conditions/sarcoidosis/10-symptoms-of-sarcoidosis/?style=quick&utm_source=adwords&adid=434578246953&ad_group_id=49173133917&utm_medium=c-search&utm_term=sarcoidosis&utm_campaign=FH-USA-Search-10-Symptoms-of-Sarcoidosis&gclid=Cj0KCQjwwtWgBhDhARIsAEMcxeCKOkEhVWWcZ3PynybQ8BRWPfH2YfUdj4QjoSnFQ9Oj6cmDxB3zVxAaAuVYEALw_wcB	https://www.merckmanuals.com/professional/pulmonary-disorders/sarcoidosis/sarcoidosis
https://www.cedars-sinai.org/programs/lung/clinical/sarcoidosis.html	https://healthprep.com/conditions/symptoms-of-sarcoidosis/?utm_source=bing&utm_medium=search&utm_campaign=566520064&utm_content=1177578007558373&utm_term=sarcoidosis&msclkid=90ed05500ff5102f180661464af3da38
https://www.pennmedicine.org/for-patients-and-visitors/patient-information/conditions-treated-a-to-z/lung-sarcoidosis	https://www.emedicinehealth.com/life_expectancy_of_a_person_with_sarcoidosis/article_em.htm
https://www.uptodate.com/contents/clinical-manifestations-and-diagnosis-of-pulmonary-sarcoidosis	https://www.nhlbi.nih.gov/health/sarcoidosis/diagnosis
https://www.uptodate.com/contents/sarcoidosis-beyond-the-basics	https://www.ncbi.nlm.nih.gov/books/NBK430687/
https://www.orpha.net/consor/cgi-bin/OC_Exp.php?Lng=GB&Expert=797	https://www.msdmanuals.com/professional/pulmonary-disorders/sleep-apnea/central-sleep-apnea
https://www.nature.com/articles/s41572-019-0096-x	https://www.livestrong.com/article/475904-the-best-foods-to-eat-if-you-have-sarcoidosis/

## References

[1] Arkema EV, Cozier YC (2018). Epidemiology of sarcoidosis: current findings and future directions. Ther Adv Chronic Dis.

[2] Hena KM (2020). Sarcoidosis epidemiology: race matters. Front Immunol.

[3] Sève P, Pacheco Y, Durupt F (2021). Sarcoidosis: a clinical overview from symptoms to diagnosis. Cells.

[4] Jain R, Yadav D, Puranik N, Guleria R, Jin JO (2020). Sarcoidosis: causes, diagnosis, clinical features, and treatments. J Clin Med.

[5] Kirkil G, Lower EE, Baughman RP (2018). Predictors of mortality in pulmonary sarcoidosis. Chest.

[6] Baughman RP, Costabel U, du Bois RM (2008). Treatment of sarcoidosis. Clin Chest Med.

[7] Baughman RP, Lower EE (2011). Who dies from sarcoidosis and why?. Am J Respir Crit Care Med.

[8] Gold DT, McClung B (2006). Approaches to patient education: emphasizing the long-term value of compliance and persistence. Am J Med.

[9] Sharp M, Brown T, Chen ES, Rand CS, Moller DR, Eakin MN (2020). Association of medication adherence and clinical outcomes in sarcoidosis. Chest.

[10] Miller TA (2016). Health literacy and adherence to medical treatment in chronic and acute illness: a meta-analysis. Patient Educ Couns.

[11] Wittink H, Oosterhaven J (2018). Patient education and health literacy. Musculoskelet Sci Pract.

[12] Bussey LG, Sillence E (2019). The role of internet resources in health decision-making: a qualitative study. Digit Health.

[13] Fox S, Jones S (2024). The social life of health information. https://www.pewresearch.org/internet/2009/06/11/the-social-life-of-health-information/.

[14] McInnes N, Haglund BJ (2011). Readability of online health information: implications for health literacy. Inform Health Soc Care.

[15] Doak CC, Doak LG, Root JH (1996). Teaching patients with low literacy skills. 2nd ed. Philadelphia, PA: JB Lippincott.

[16] (2024). United States Government Accountability Office: report to congressional requesters. Medicare: communications to beneficiaries on the prescription drug benefit could be improved. https://www.gao.gov/assets/gao-06-654.pdf.

[17] Walsh TM, Volsko TA (2008). Readability assessment of internet-based consumer health information. Respir Care.

[18] Akinleye SD, Garofolo-Gonzalez G, Montuori M, Culbertson MD, Hashem J, Edelstein DM (2018). Readability of the most commonly accessed online patient education materials pertaining to pathology of the hand. Hand (N Y).

[19] Mc Carthy A, Taylor C (2020). SUFE and the internet: are healthcare information websites accessible to parents?. BMJ Paediatr Open.

[20] Eysenbach G, Köhler C (2002). How do consumers search for and appraise health information on the world wide web? Qualitative study using focus groups, usability tests, and in-depth interviews. BMJ.

[21] Murray TE, Mansoor T, Bowden DJ, O'Neill DC, Lee MJ (2018). Uterine artery embolization: an analysis of online patient information quality and readability with historical comparison. Acad Radiol.

[22] O'Neill SC, Baker JF, Fitzgerald C, Fleming C, Rowan F, Byrne D, Synnott K (2014). Cauda equina syndrome: assessing the readability and quality of patient information on the Internet. Spine.

[23] Zhou S, Jeong H, Green PA (2017). How consistent are the best-known readability equations in estimating the readability of design standards?. IEEE Trans Professional Commun.

[24] Spadaro DC, Robinson LA, Smith LT (1980). Assessing readability of patient information materials. Am J Hosp Pharm.

[25] Zheng J, Yu H (2018). Assessing the readability of medical documents: a ranking approach. JMIR Med Inform.

[26] Devgire V, Martin AF, McKenzie L, Sandler RD, Hughes M (2020). A systematic review of internet-based information for individuals with Raynaud's phenomenon and patients with systemic sclerosis. Clin Rheumatol.

[27] Reynolds M, Hoi A, Buchanan RR (2018). Assessing the quality, reliability and readability of online health information regarding systemic lupus erythematosus. Lupus.

[28] Özduran E, Hanci V (2022). Evaluating the readability, quality and reliability of online information on Behçet's disease. Reumatismo.

[29] Vargas CR, Ricci JA, Chuang DJ, Lee BT (2016). Online patient resources for liposuction: a comparative analysis of readability. Ann Plast Surg.

[30] Schmitt PJ, Prestigiacomo CJ (2013). Readability of neurosurgery-related patient education materials provided by the American Association of Neurological Surgeons and the National Library of Medicine and National Institutes of Health. World Neurosurg.

[31] Sare A, Patel A, Kothari P, Kumar A, Patel N, Shukla PA (2020). Readability assessment of internet-based patient education materials related to treatment options for benign prostatic hyperplasia. Acad Radiol.

[32] Badarudeen S, Sabharwal S (2010). Assessing readability of patient education materials: current role in orthopaedics. Clin Orthop Relat Res.

[33] DuBay WH (2024). The principles of readability. Online Submission.

[34] Hickey KT, Masterson Creber RM, Reading M, Sciacca RR, Riga TC, Frulla AP, Casida JM (2018). Low health literacy: implications for managing cardiac patients in practice. Nurse Pract.

[35] Williams MV, Davis T, Parker RM, Weiss BD (2002). The role of health literacy in patient-physician communication. Fam Med.

[36] (2024). Language use in the United States: 2019. https://www.census.gov/content/dam/Census/library/publications/2022/acs/acs-50.pdf.

